# Challenges with hypertension self-care among survivors of adverse childhood experiences

**DOI:** 10.1016/j.ssmqr.2022.100065

**Published:** 2022-03-05

**Authors:** Carmen Alvarez, Nadia Andrade, Jagriti “Jackie” Bhattarai, Robert Okyere, Lisa A. Cooper

**Affiliations:** a Johns Hopkins University School of Nursing, 525 North Wolfe Street, Baltimore, MD, 21205, USA; b Johns Hopkins University School of Medicine, Department of Medicine, Division of General Internal Medicine, 2024 East Monument Street, Suite 2-500, Baltimore, MD, 21287, USA; c Johns Hopkins Center for Health Equity, 2024 East Monument Street, Suite 2-500, Baltimore, MD, 21287, USA; d Johns Hopkins Bayview Medical Center, 4940 Eastern Ave, Baltimore, MD, 21224, USA; e Johns Hopkins Bloomberg School of Public Health, Department of Health, Behavior and Society, 624 North Broadway, Baltimore, MD, 21205, USA

**Keywords:** Hypertension, Adverse childhood experiences, Coping, Attitudes

## Abstract

Uncontrolled hypertension remains a serious public health problem. Despite the long-term negative effects of adverse childhood experiences (ACEs) on health and health behaviors, few studies have explored how to support ACE survivors with uncontrolled hypertension. We conducted semi-structured individual interviews with 24 diverse patients to understand how ACE survivors may manage their hypertension compared to those who had not experienced ACEs. Using qualitative descriptive methods we categorized participants based on ACE experience: non ACEs, ACEs with sexual abuse, and ACEs without sexual abuse. Our thematic analysis revealed 5 themes: stress, childhood experiences, social support, experiences with health care, and attitudes about self-care. These themes revealed more about antecedents to health behaviors than about actual management of hypertension. Although all participants experienced challenges with healthy lifestyle changes, comments from those who did not experience ACEs reflected a more positive attitude about self-care, and adaptive strategies for overcoming challenges related to hypertension management. ACE survivors reported more depressive symptoms, maladaptive strategies, and negative experiences with health care which negatively impacted their disease management. ACE survivors may need additional resources for depression management and coping strategies for self-care—all of which may improve health behavior and blood pressure control.

Globally, hypertension is the strongest modifiable risk factor for cardiovascular disease and its sequelae (Olsen et al., 2016). In the US, approximately 1 in 3 adults (32%) have hypertension and despite increased hypertension awareness and treatment options, less than half (48.3%) of adults with hypertension have their blood pressure controlled to less than 140/90 mmHg (Fryar et al., 2017). To address this global issue, researchers have called for a life-course approach for primary and secondary prevention of hypertension and to optimize quality of life (Olsen et al., 2016). A life-course approach refers to the study of long-term effects of physical and social exposures during gestation, childhood, adolescence, young adulthood, and later adult life on an individual’s health and the health of populations across generations (Ben-Shlomo et al., 2016). The life-course approach is useful for uncovering and understanding modifiable factors that can impact blood pressure control.

A growing body of research connects a history of toxic stress or adverse childhood experiences (ACEs) to increased lifetime risk for cardiovascular disease (Merrick et al., 2019; Petruccelli et al., 2019). Specifically, compared to those who do not report ACEs, experiencing 4 or more ACEs almost doubles the risk of developing heart disease (Godoy et al., 2021). ACEs refer to sources of toxic stress or trauma in childhood to include: multiple types of abuse (physical, psychological, and sexual), physical or emotional neglect, household dysfunction (witnessing domestic violence, living with an adult with mental illness, parental divorce, mental illness, criminal behavior or loss) (Felitti et al., 2019), bullying, and exposure to community violence (Finkelhor et al., 2015). Given the long-term negative effects of ACEs, failure to identify survivors and their additional needs may contribute to delayed detection and undertreatment of hypertension. Despite the association between a history of ACEs and chronic disease, few studies have explored how to support survivors of ACEs with uncontrolled hypertension.

There are multiple pathways through which ACEs may impact hypertension: biological, psychosocial, and behavioral (Su et al., 2015). This study focuses on modifiable psychosocial and behavioral factors. Many survivors of ACEs thrive without mental health challenges such as depression and anxiety, therefore differences in cognitive processing about experienced adversity are suggested to explain some of the variation in psychological well-being among survivors (Lazarus, 1993; Lazarus & Folkman, 1987). It has been suggested that early life chronic adversity (e.g. poverty, discrimination) and toxic stress from ACEs disrupt brain development, cognitive function, and ultimately psychosocial resources such as social support, sense of control, and self-esteem that are essential for managing stress (Hager & Runtz, 2012; Nurius et al., 2013). According to the Conservation of Resources Theory (Hobfoll et al., 2003), having these depleted psychosocial resources contributes to perceptions of stress. Stress management strategies are often categorized as problem-focused (strategies directed at the stressor) or emotion-focused (strategies directed at emotions resulting from the stress) (Biggs et al., 2017). Coping styles are also associated with severity of hypertension, for example Casagrande et al., 2019, found that emotion-focused coping was associated with uncontrolled hypertension. Compared to those who have not experienced ACEs, ACE survivors have greater levels of perceived stress (Mersky et al., 2017) and emotion-focused coping (Spaccarelli, 1994), and lower perceived social support (Cheong et al., 2017). Among survivors with hypertension, emotion-focused coping strategies may interfere with blood pressure control by exacerbating stress and impeding self-care through compensatory but maladaptive behaviors such as smoking, physical inactivity, poorer stress management, and medication non-adherence. ACE survivors are also at increased risk for depression—another risk factor for uncontrolled hypertension (Meng et al., 2012; Rubio-Guerra et al., 2013). In sum, ACE survivors with hypertension may be at increased risk for uncontrolled blood pressure and warrant greater attention.

In this study, we focused on survivors of ACEs with uncontrolled hypertension. The purpose of this study was to understand how ACE survivors may manage their hypertension compared to those who have not experienced ACEs. Prior research links history of child sexual abuse to greater depression, reports of poorer health, and greater healthcare utilization, compared to those who experienced non-sexual forms of child abuse (Irish et al., 2010; Wilson, 2010). As such, in this study, we also sought to capture experiences of survivors of child sexual abuse. Given that ACE survivors have poorer health outcomes and health behaviors, we hope to inform how to best support survivors for optimal blood pressure control. Informed patients who are confident about managing their chronic disease are more likely to report regular exercise, better stress management, greater care with dietary intake, and medication compliance (Hibbard et al., 2013). Therefore, as clinicians, we have an opportunity to facilitate these health behaviors through building the confidence and skills ACE survivors may need for optimal blood pressure control.

## 1. Methods

### 1.1. Parent study

This qualitative descriptive work was part of the RICH LIFE (Reducing Inequities in Care of Hypertension: Lifestyle Improvement for Everyone) project, a pragmatic trial designed to compare the effectiveness of enhanced standards of care with a clinic-based multilevel intervention that includes behavior change counseling by a nurse care manager and, if needed, additional support from a community health worker or consultation from a group of specialists to improve blood pressure control (Cooper et al., 2020). The RICH LIFE project included partnerships with multiple federally qualified health centers (FQHC) and private clinics throughout Maryland and Pennsylvania. FQHCs are primary care safety-net clinics located in underserved areas throughout the U.S. and are intended to address health disparities and serve primarily low-income and underinsured populations (Health Resources and Services Administration, 2019). The FQHCs in this study were located in both urban and rural areas. The private clinics were located mainly in suburban settings and served patients from the spectrum of socioeconomic strata.

Eligibility criteria for the parent study included: (1) ≥ 21 years of age, (2) non-Hispanic white, non-Hispanic African American, or Hispanic ethnicity, and (3) a diagnosis of hypertension with at least one of the following cardiovascular risk factors – diabetes mellitus, hyperlipidemia, coronary health disease, current tobacco smoker, and/or diagnosis of depression; more study details are described elsewhere (Cooper et al., 2020). Eligible participants were invited to participate in the RICH LIFE project with a copy of oral consent. Those who expressed interest in the study were consented and completed a telephone baseline survey, which, among other measures (age, race/ethnicity, systolic and diastolic blood pressure, depression), included the Adverse Childhood Experiences questionnaire and the Personal Health Questionnaire (PHQ-8).

The 10-item ACE questionnaire is a widely used tool for assessing history of different types of childhood adversity such as: physical, emotional, and sexual abuse; physical and emotional neglect; and household dysfunction including living with adults with mental illness and/or witnessing domestic violence (Dube et al., 2004). Participants responded yes or no to each question, responses were then summed for a total score ranging from 0 to 10. The PHQ-8 is also an established clinical tool used to assess depression and does not include assessment for suicidal ideation (Wu et al., 2019). The tool assesses for frequency (0 – not at all, 3 – nearly every day) of symptoms commonly experienced with depression (e.g. trouble falling or staying asleep, or poor appetite or overeating). Higher scores on the PHQ-8 indicate greater severity of depression (α = 0.84).

### 1.2. Procedures for sub-study

The Johns Hopkins School of Medicine institutional review board approved this study and all procedures. For this sub-study we used qualitative descriptive methods because we wanted to explore how survivors of ACEs managed their hypertension compared to those who did not report experiencing ACEs (Neergaard et al., 2009; Sandelowski, 2000). Our qualitative descriptive methods included: (1) purposive sampling to obtain perspectives from three groups: those who had not experienced ACEs (non-ACEs), and survivors of ACEs with and without sexual abuse; (2) semi-structured interviews to learn about how participants managed their hypertension, and (3) thematic analysis to identify themes and patterns across the groups (Braun & Clarke, 2006; Castleberry & Nolen, 2018).

#### 1.2.1. Sampling strategy

Our sampling strategy started with a list of participants who completed the baseline survey which included the ACEs questionnaire. We categorized this list based on our three groups of interest: those who had not experienced ACEs (non-ACEs), and survivors of ACEs with and without sexual abuse. Although our exposure variable of interest was ACE experience, we wanted to reflect the diversity of the parent study sample in each of the sub-study groups. Thus, we implemented stratified sampling to have representation by gender (men and women), race/ethnicity (Black, White, Hispanic), and from FQHCs (both rural and urban) and private clinics.

#### 1.2.2. Data collection approach

Because the parent study included clinics from across Maryland and Pennsylvania, it was not feasible to conduct in-person interviews will all participants. Therefore, to utilize the same method with all participants we conducted phone interviews. We anticipated that roughly eight participants from each group would suffice to reach data saturation – a point at which we start to hear similar responses from participants (Saunders et al., 2018). Two authors (both clinicians and nurse researchers) then called participants and invited them to participate in a sub-study about understanding how participants manage their blood pressure. Both of the interviewers had prior experience with qualitative research, with one interviewer having more years of experience and practice. The interviewers reviewed and discussed the interview guide to ensure that both interviewers understood the intent of the questions. Also, as interviews were conducted, the interviewers critically reviewed the audio-recording to identify areas of improvement and if interview questions needed re-phrasing. The authors conducted the phone interviews which ranged from 20 to 75 min (average – 30.28 min) in duration. We did not identify any questions that need clarification. Table 1 lists the semi-structured interview questions. Given that all participants had already completed the ACEs questionnaire, we did not directly inquire about childhood adversities. Interviews were conducted between June 2018 and March 2019, and participants were compensated $60.

### 1.3. Data analysis

Part of our purposive sampling was to include those who did and did not report experiencing ACEs in order to explore for differences between the groups (Morse, 2015). Transcripts were entered into F4analyse software (n.d.) for coding and collocation of quotes. For thematic analysis, we reviewed the domains and descriptive codes for patterns and commonalties. We then constructed a table organizing participants by ACE category, and then by domains with their coded responses. We independently read and re-read the tables, noted emerging themes, and then discussed them as a group (all members involved in coding) until we reached agreement about the themes, potential connections between the themes, corresponding codes, and illustrative texts (Morse, 2015). During this process of iterative analysis and identifying themes, redundancies in domains and themes became apparent. Given that no new themes were identified within and between the ACE categories, we assert that inductive thematic saturation was achieved (Saunders et al., 2018). We used descriptive analyses to describe sample characteristics.

### 1.4. Maintenance of rigor

We implemented multiple strategies to ensure the rigor of data collection and analysis. All interviews were audio-recorded and transcribed verbatim by a professional third party. Transcripts were reviewed for accuracy by multiple authors. We also developed a coding system for consistency of coding between coders. Three authors independently read and open-coded the transcripts. The codes were inductive where we developed our codes based on participant comments. The authors then met to compile and discuss the descriptive codes. Next, we sorted the codes into domains and developed a joint codebook for consistency and focused coding. While independently coding the interviews, the authors met on multiple occasions to review coding of transcripts for coding agreement and discuss emerging themes. Where there was disagreement a third author served as the arbiter. To foster reflexivity, we also discussed notes from our memos, as well as, noted our reflections and ideas about – among other things – potential explanations for participants’ perceptions and reactions to various situations (Miles & Huberman, 1984). Data collection and analysis occurred in an iterative fashion (Boeije, 2002).

## 2. Results

Participant baseline demographics, blood pressure levels (obtained from their most recent visit in the electronic health record), depression levels on the Patient Health Questionnaire, and mean number of ACEs are displayed in Table 2. Participants were on average 56 years old, mostly female (58.3%), and Black and White participants were equally represented. With the exception of one person, all participants were on prescribed antihypertension medications at the time of the interview. The only major difference between the three groups was depression levels, non-parametric independent-samples median test indicated that ACE survivors had higher scores for depression symptoms (M(SD) = 7.7 (3.6) ACEs no sexual abuse; M(SD) = 5 (6.6) sexual abuse) compared to the non-ACEs group (M(SD) = 1.1 (1.4), p < .05).

We reached data saturation with our sample and identified the following themes from the descriptive qualitative analysis: (a) stress (b) childhood experiences, (c) social support, (d) experiences with health care, and (e) attitudes about self-care.

### 2.1. Similarities between Non-ACE group and ACE survivors

#### 2.1.1. Stress

Stress emerged as a major theme across all groups. We identified four sub-themes for stress: reasons for uncontrolled blood pressure, challenges with lifestyle changes, community environment, and coping strategies. Almost all participants identified different types of stressors as a main reason for why their blood pressure was uncontrolled and as a contributor to making lifestyle changes challenging. We also learned about the coping strategies used to manage stress.

##### 2.1.1.1. Reasons for uncontrolled blood pressure.

When reflecting on why they had uncontrolled blood pressure, participants described an array of factors that they considered to be stressful. Stressors included daily challenges such as work-related issues, and major life events such as the death of family members. For example, one ACE survivor who owned a farm, shared how a “build up” of “little” concerns about the farm (e.x. stock prices for his product and timing of planting) contributed to his persistent worry, feeling stressed, and ultimately uncontrolled blood pressure. Another woman (non-ACEs) attributed her high blood pressure to the many activities she had to do to manage her health. This woman felt like the self-management activities such as setting up appointments, verifying medications, and having the money to complete these activities was overwhelming and required a lot of work. Regarding major life events, another participant (non-ACEs) described how being a caregiver for her ill mother prohibited her from exercise and maintaining a healthy diet. Being a caregiver resulted in her gaining over ten pounds a year for several years, which contributed to her uncontrolled hypertension.

##### 2.1.1.2. Challenges with lifestyle changes.

For many participants, implementing and sustaining healthy lifestyle behaviors was challenging due to other daily activities which were also sources of stress. Behaviors they were trying to implement included stress management, decreasing smoking, exercise, and being more mindful of their dietary intake – including reading food labels and measuring portion sizes. For example, one woman explained her challenges with stress management and how it impacted other health behaviors. She said:

I think the hardest one was the managing the stress because if my stress level was really high, I wouldn’t be able to sleep good, and then the more tired I got, the more junk food I was eating and just not doing anything. (Female 5, ACEs)

Another ACE-survivor shared how finding the time to exercise or eat healthy was often a challenge due to her commute and children’s schedule. She explained, “… you don’t have time to sit down and eat, or prepare a healthy breakfast, or a lunch; you just grab something on the run.” She further explained that by the time she was done with all of her responsibilities it is bedtime and she does not have the energy to exercise. Participants who had not experienced ACEs reported similar challenges with implementing and maintaining lifestyle changes for blood pressure control.

##### 2.1.1.3. Community environment.

While we focused on differences between ACE categories, we identified similar comments among those residing in urban settings. Urban participants shared multiple concerns about their community including: homicide, drug dealing, vandalism of personal property, and blight. One woman shared that what made these experiences more frustrating was the lack of recourse.

I don’t know how many times my car has been ruffled through and things stolen out of it. When I call the police, this is what I’m told. Oh, you live in the city. You have to expect that. (Female 7, ACEs).

These types of events not only contributed to stress that could impact blood pressure, but was also considered a reason for a hypertension diagnosis by some participants. Three Black female participants who resided in urban areas reported that they became hypertensive after their sons were killed in gun violence. In contrast, regardless of race or gender, participants living in suburban and rural areas described environments in which they could leave their front doors unlocked, and go out for walks on trails.

### 2.2. Differences between Non-ACE group and ACE survivors

#### 2.2.1. Stress: coping strategies

During discussions about lifestyle challenges and stressors, participants revealed their coping strategies. We identified more problem-focused strategies among the non-ACE participants compared to ACE survivors. Participants in the non-ACEs group shared strategies they used to minimize stress and adhere to medications and lifestyle changes. For example, one woman shared how she started doing chores over the course of the week so that she would not feel overwhelmed trying to complete them in a day or over the weekend. Another shared how her and her husband found exercises to do inside the house if was too cold to walk outside. Even when dealing with pain, one patient identified ways to remain active.

During the week, I may have to walk to go to get my medicine, which gives me some exercise. Even though I can take the MTA [state Transit Administration], I try to get some exercise in because of my weight. I’m trying to lose some …. Even though I’m in pain, I still walk to try to keep my other joints from atrophying. (Female 11, non-ACEs)

Although some ACE survivors also shared problem-focused strategies, they mostly shared emotion-focused strategies. These strategies included isolating themselves when stressed, yelling, smoking, or working a lot of hours so that there would not be any time to think about one’s feelings or deal with ongoing stressors. One ACE survivor was at the point of inaction for her blood pressure because the provider did not prescribe her preferred medication and she did not want to restart with different medications. She said, “I’m frustrated. I know most likely they’ll start at the beginning again.” Because she did not feel like going through the process of restarting antihypertension medication and experiencing the potential side effects, she opted to remain with her blood pressure uncontrolled and took her medications only when she felt her blood pressure was too high.

#### 2.2.2. Childhood experiences

Seven ACE survivors (6 women, 1 man) recounted negative experiences from childhood, including: being in the courtroom for parental divorce, having a sole parent die, sexual abuse, and physical and emotional abuse from an alcoholic mother. One male recounted multiple adversities that he experienced as a child:

My daddy was a woman beater. He just abandoned us …. my granddad was there and all but he was an alcoholic. He wasn’t mean. He was a very nice man, nevertheless he killed hisself and we got to see that and it was horrific.

In contrast, negative childhood experiences were not reported by participants in the non-ACEs group. In talking about themselves and how they grew up, participants from all groups reflected on positive experiences. Participants recalled enjoying spending time with family, living in friendly neighborhoods where your could borrow a cup of sugar, and growing up at a time when you could be out all day playing and just had to be home by sunset for dinner.

#### 2.2.3. Social support

Notable among the non-ACE group was their mention of social support. These “non-ACE” participants commented more on quality family relationships in which they enjoyed spending time with family members and, also, received encouragement and instrumental support that promoted healthy behavior change. For instance, one woman shared how exercising with her family helped to keep her engaged and active. One participant shared how her children routinely checked-in on her, and made sure that they brought her groceries or anything else she may have needed. For another woman, it was her husband encouraging the behavior change.

More non-ACE participants than those who experienced ACEs also talked about close friendships and being involved in their communities. Activities with friends ranged from sitting on a porch for fellowship, to going on daily walks. For another woman, despite living in pain, she was still committed to serving and being part of her community.

I work with the children at my church and also help in the community while I’m not working. Still volunteering because there’s so many children that are in need. I find even with my sickness, I can still [be helpful] there’s a lot of children that need hugs and talking to. I enjoy doing that while I’m recuperating. (Female 11, non-ACEs)

ACE survivors tended to report more instrumental social support rather than the emotional support expressed by their counterparts who did not report ACEs. The female ACE survivors in particular shared how their grandchildren or neighbor would help them with chores such as walking the dog, cleaning, etc.

Some ACE survivors also seemed to suggest that they did not have any social support despite having family members available to them. For example, some survivors commented “… I got nobody. Nothing here, it’s just me,” (Male 2) and “Everybody says they’ll help me. Nobody comes to help me …. I don’t ask nobody for nothin’” (Male 3). On the other hand, one survivor, who described having frequent positive interaction with family, shared the following: “… I have a really good attitude about life. I’m a very optimistic person … I always look for the good impact” (Male 4).

#### 2.2.4. Experiences with health care

Most participants reported positive experiences with healthcare, particularly their interactions with providers. Good providers were consistently described as being active listeners, available to respond to requests, knowledgeable, and encouraging for behavioral change. One participant shared:

…. she sits down, she talks to me; she explains everything that’s going on and answers my questions. If there’s a question that she thinks I should ask, she would ask me? It’s like she can almost—there’s something you’re forgetting to ask me, you need to remember what it is … It doesn’t matter what the time of the day is, that I feel as though it’s not right, I can call her …. (Female 9, non-ACEs)

Participants in all three groups described experiences similar to this woman.

Negative experiences with healthcare were also identified across all groups; however, reports of negative experiences were mainly from survivors of child sexual abuse. These negative experiences were often about confusion around diagnoses, disagreements or misunderstanding on plans of care, and not feeling cared for by the provider. For some, these experiences were bad enough to prevent the participant from returning for care. One woman described her experience seeking care for depression as “the worst thing I ever did in my life …” She described the provider as being very abrupt to the point where she left the visit and never sought care again for her depression.

Another shared how the provider not acknowledging his efforts for weight loss was off-putting and caused him not to follow up with the provider.

… I had lost a lot of weight. I had lost 25 pounds or something like that. I’d really worked at it over a period of time. I sat there in the office. This guy never looked at me and says—and he even had the chart in front of him. Could’ve said, “Oh, you lost weight.” He never said that. He never noticed that I’d lost weight. I’m looking at him and going, “Why am I here?” (Male 4, ACEs)

Two participants in the non-ACE group also shared some negative experiences. However, compared to survivors of ACEs, non-ACE participants described frustrations with healthcare processes such as following up on prescriptions and referrals, rather than discontent with actual providers.

#### 2.2.5. Attitudes about self-care

Although participants across all groups were experiencing challenges with lifestyle changes, we found that non-ACE participants were more likely to have a positive outlook on their challenges and believed that they could overcome them. Participants in the non-ACEs group talked about “looking things up” for a better understanding of how to manage their blood pressure, and “working slowly” towards changes. One woman (non-ACEs) discussed how she prioritized physical activity despite her pain – “Even with my cane, I still try to go to a dance class once a week.” Examples of these positive attitudes were not identified among ACE survivors. Instead, some suggested that their hypertension was not really something in their control and therefore their behavior would not do much to change their blood pressure. For example, one male, ACE survivor stated, “I’m black and people have hypertension at times no matter what they do …”

## 3. Discussion

When considering a life-course approach for adults with hypertension, the results of this qualitative study suggest that even in a sample of participants with uncontrolled hypertension, the ACE survivors may have different needs for hypertension self-management compared to those who have not experienced ACEs. To our knowledge, this is the first study that examined how ACE survivors and those who have not experienced ACEs may manage their condition. Our findings contribute to the growing body of literature about the long-term negative impact of ACEs on health.

In this study, participants revealed more about antecedents to health behaviors than management of their condition. Stress management, changing dietary habits, and increasing physical activity were challenges across all groups; it makes sense that most were experiencing some sort of challenge given that uncontrolled hypertension was an inclusion criterion. We identified some differences in attitudes about these challenges between ACE survivors and the non-ACEs group. Those in the non-ACEs group believed that they could overcome their respective challenges and articulated steps towards making changes. Such a positive attitude and belief in one’s ability to manage blood pressure has been associated with self-care motivation, which in turn was associated with self-care behaviors for blood pressure control (Peters & Templin, 2010; Warren-Findlow et al., 2012). Therefore, while not directly related to self-care behaviors, positive attitudes and beliefs towards self-care behaviors are critical components of the pathway for supporting behavioral changes, and survivors of ACEs may need more support than those without such a history.

Results from this study align with stress process frameworks (Hobfoll et al., 2003; Lazarus & Folkman, 1987; Pearlin et al., 1981), where ACE survivors were more likely to be depressed, report maladaptive coping strategies, and have less positive attitudes about self-care. ACEs have been linked to negative cognitions about the self, neuroticism, and emotion-focused coping (Mc Elroy & Hevey, 2014; Mosley-Johnson et al., 2019). Our study provides additional context for these findings. The Parental Acceptance-Rejection Theory suggests that child maltreatment can negatively impact personality development, coping styles and ultimately one’s outlook on life (Rohner et al., 2005), and the development of healthy relationships which are essential for forming a social support system (Hill et al., 2010; Nurius et al., 2012). In this study, we observed more comments about social support from family and friends in the non-ACEs group compared to the ACEs group. Even survivors with immediate family seemed to perceive themselves as being alone. This is disconcerting given the benefits of social support on mental health (Evans et al., 2013), perceived stress (Cheong et al., 2017), medication adherence, and uncontrolled hypertension (Cornwell & Waite, 2012).

Positive experiences with healthcare is another important component of hypertension control. While most participants reported positive experiences with healthcare, ACE survivors recounted more negative experiences that affected their engagement with care. One participant remained disengaged and uncontrolled. ACE survivors also reported negative experiences that were characteristically different from the non-ACE participants’ experiences. Non-ACE participants described experiences with healthcare system processes rather than with individual providers. This may be explained in part by the emotion dysregulation often associated with ACEs; this increased vulnerability to react with negative emotions may also contribute to ACE survivors’ reactions and negative perceptions in situations that are not proceeding as expected (Dvir et al., 2014). Even though the non-ACE participants described some frustrations with healthcare, their feelings of discontent did not become a barrier to care.

Similar to other studies (Jones et al., 2018; Logan-Greene et al., 2014), ACE survivors in this sample reported depressive symptomatology. Depressive symptoms have been linked to poorer self-care, emotion-focused coping, low perceptions of social support (Artinian et al., 2006; Rueda & Pérez-García, 2006), and poorer medication adherence among patients with uncontrolled blood pressure (Hooper et al., 2016). These findings underscore the importance of screening for depression, particularly among patients who are ACE survivors with uncontrolled hypertension.

Regardless of ACE category, our participants discussed community environment as a source of stress. Other studies have also identified how community violence and community disorder negatively impact self-management behaviors such as medication adherence (Lewis et al., 2010; Alvarez et al., 2021). Indeed, issues of personal safety and crime have been associated with depression (Barnett et al., 2018). This raises the issue of social determinants of health (SDoH), and the need to move beyond individual-level factors and address community-level issues for improved population health. Another SDoH that did not emerge as an issue was perceived discrimination. However, the reported community violence and disorder may be an indicator of structural racism that contribute to neighborhood disadvantage and ultimately health behavior and health (Scribner et al., 2017).

Of note in our sample was the mention of positive childhood experiences. Recent work by Bethell et al., 2019 highlight the benefit of “positive childhood experiences” for decreasing risk for poor mental health or depression among survivors of ACEs. Given our study design and sample size, we were not able to assess to what extent positive childhood experiences impacted antecedents for self-management (i.e. social support and attitudes towards care).

### 3.1. Study limitations

We focused on how ACE survivors were managing their hypertension compared to those who had not experienced ACEs in a sample in which all participants were uncontrolled. It would have been of value to also include responses from participants who were controlled to obtain insights into how ACE survivors were able to obtain control and whether they too were challenged with negative experiences in healthcare, social support, and depression. Further, we only assessed for the traditional ACEs – including asssessments for expanded ACEs may have yielded yet another category of participants for whom we may have identified different experiences. Also, eventhough ACE survivors are at increased risk for cardiovascular disease, resiliency may mitigate the negative impact of early adversity (Ross et al., 2020). Thus, interviewing participants over a period of time would have been useful to gain insights about resiliency among ACE survivors and learn how and if survivors compared to non-ACEs participants were able to obtain blood pressure control. Finally, our sample comprised primarily Black and White participants; including participants from other cultures would also be valuable as our patient populations are becoming increasingly diverse.

### 3.2. Relevance for clinical practice

Our findings are an important contribution to the literature as they highlight potential points of intervention for ACE survivors with uncontrolled hypertension. Clearly absent from our findings was participants’ misinformation about hypertension; in other words, participants’ comments did not suggest that they were unaware of ideal health behavior for hypertension control. From a practice perspective, the tendency is to “educate” and “inform” patients about how to better manage their hypertension. Although future studies are needed to quantify the level of self-management skills and behaviors in this population, our findings suggest that uncontrolled hypertension among ACE survivors may not be an issue of education, but rather of low psychosocial resources to address stress and depression. Aside from pharmacological treatment, multiple psychotherapies have been effective for depression (Bell & D’Zurilla, 2009; Ekers et al., 2014; Lopez & Basco, 2015) and hold potential for improving self-esteem, perceived social support, and coping styles in ACE survivors. In addition to psychotherapy and pharmacological treatment, there are primary care provider behaviors that may be beneficial to ACE survivors, such as being intentional about partnering with patients for their care, brainstorming with them on strategies for behavioral change, and showing care for patients as individuals (Alvarez et al., 2016).

### 3.3. Future research

Our findings highlight the need to identify which types of support may be most effective for ACE survivors with uncontrolled hypertension. Next steps for research should include examining the effectiveness of multilevel interventions for sustained blood pressure control among ACE survivors. Models of care that address mental health comorbidities, as well as, provide ongoing non-medical support to address social determinants of health may be ideal for ACE survivors who are more likely to be depressed and feel overwhelmed with managing daily challenges.

## 4. Conclusions

In summary, we learned in this novel study that ACE survivors with uncontrolled blood pressure may warrant more additional care than non-ACE patients with uncontrolled blood pressure. Therefore, healthcare providers may want to consider assessing for ACEs, particularly among patients with uncontrolled chronic disease, as they may warrant more holistic care. ACE survivors may need additional resources to help them manage their depressive symptoms, improve their social support, coping strategies, and attitudes about their self-care—all of which may positively impact their self-care behaviors and ultimately their blood pressure control.

## Figures and Tables

**Table 1:** Semi-structured interview questions.

1. Please tell us a little about yourself.
How do you spend your day? Tell me about your family life.
2. When were you first diagnosed with hypertension?
What was this experience like for you? How did you feel about being told that you had hypertension?
3. What types of things do you do now to manage your hypertension?
How do you think you manage your hypertension now, compared to when you were first diagnosed? What do you believe sometimes causes your blood pressure to be high?
4. How would you describe your relationship with your healthcare providers?
What do you like about how your health care provider cares for you? What do you not like about how your health care provider cares for you?

**Table 2:** Characteristics of study sample.

	Total Sample N = 24	No Adverse Childhood Experiences n = 8	Adverse Childhood Experiences without Child Sexual Abuse n = 7	Child Sexual Abuse n = 9
Age, mean	56.96 (9.72)	62 (10)	56.3 (7.5)	53 (9.9)
Gender, n(%)				
Male	10 (41.7)	3 (12.5)	3 (12.5)	4 (16.7)
Female	14 (58.3)	5 (20.8)	4 (16.7)	5 (20.8)
Race/Ethnicity, n(%)				
White	12 (50)	3 (12.5)	5 (20.8)	4 (16.7)
Black or African American	11 (45.8)	4 (16.7)	2 (8.3)	5 (20.8)
Hispanic	1 (4.2)	1 (4.2)	0	0
Geographic Location				
Urban	8 (33.3)	2 (8.3)	1 (4.2)	5 (20.8)
Suburban	11 (45.8)	4 (16.7)	4 (16.7)	3 (12.5)
Rural	5 (20.8)	2 (8.3)	2 (8.3)	1 (4.2)
Years of Education, mean	13.9 (2.9)	14.13 (2.5)	14.4 (4)	13.5 (3)
Systolic Blood Pressure, mean	153.75 (10.9)	156.5 (11.5)	150.4 (10.8)	153.9 (11.1)
Diastolic Blood Pressure, mean	91.8 (9.3)	87.6 (10.2)	91.8 (9.6)	95.4 (7.4)
Patient Health Questionnaire-8, mean	4.5 (5.1)	1.13 (1.4)	7.7 (3.6)	5 (6.6)
Mean number of ACEs			6.2 (3.2)	4.2 (2.7)

## References

[R1] Alvarez C, Greene J, Hibbard J, & Overton V. (2016). The role of primary care providers in patient activation and engagement in self-management: a cross-sectional analysis. BMC Health Services Research, 16(85), 1–8. 10.1186/s12913-016-1328-3 26969293 PMC4788946

[R2] Alvarez C, Hines AL, Carson KA, Andrade N, Ibe C, Marsteller JA, & RICH LIFE Project Investigators. (2021). Association of perceived stress and discrimination on medication adherence among diverse patients with uncontrolled hypertension. Ethnicity & Disease, 31(1), 97–108. 10.18865/ed.31.1.97 33519160 PMC7843046

[R3] Artinian NT, Washington OGM, Flack JM, Hockman EM, & Jen K-LC (2006). Depression, stress, and blood pressure in urban African-American women. Progress in Cardiovascular Nursing, 21(2), 68–75. 10.1111/j.0889-7204.2006.04787.x 16760688

[R4] Barnett A, Zhang CJ, Johnston JM, & Cerin E. (2018). Relationships between the neighborhood environment and depression in older adults: A systematic review and meta-analysis. International Psychogeriatrics, 30(8), 1153–1176. 29223174 10.1017/S104161021700271X

[R5] Bell AC, & D’Zurilla TJ (2009). Problem-solving therapy for depression: A meta-analysis. Clinical Psychology Review, 29(4), 348–353. 10.1016/j.cpr.2009.02.003 19299058

[R6] Bethell C, Jones J, Gombojav N, Linkenbach J, & Sege R. (2019). Positive childhood experiences and adult mental and relational health in a statewide sample: Associations across adverse childhood experiences levels. JAMA Pediatrics, 173(11). e193007-e193007. 10.1001/jamapediatrics.2019.3007 PMC6735495 31498386

[R7] Ben-Shlomo Y, Cooper R, & Kuh D. (2016). The last two decades of life course epidemiology, and its relevance for research on ageing. International Journal of Epidemiology, 45(4), 973–988. 10.1093/ije/dyw096 27880685 PMC5841628

[R8] Biggs A, Brough P, & Drummond S. (2017). Lazarus and Folkman’s psychological stress and coping theory. The handbook of stress and health: A guide to research and practice, 351–364.

[R9] Boeije H. (2002). A purposeful approach to the constant comparative method in the analysis of qualitative interviews. Quality and Quantity, 36(4), 391–409.

[R10] Braun V, & Clarke V. (2006). Using thematic analysis in psychology. Qualitative Research in Psychology, 3(2), 77–101.

[R11] Casagrande M, Boncompagni I, Mingarelli A, Favieri F, Forte G, Germano R, Germano G, & Guarino A. (2019). Coping styles in individuals with hypertension of varying severity. Stress and Health, 35(4), 560–568. 31397061 10.1002/smi.2889

[R12] Castleberry A, & Nolen A. (2018). Thematic analysis of qualitative research data: Is it as easy as it sounds? Currents in Pharmacy Teaching and Learning, 10(6), 807–815. 30025784 10.1016/j.cptl.2018.03.019

[R13] Cheong EV, Sinnott C, Dahly D, & Kearney PM (2017). Adverse childhood experiences (ACEs) and later-life depression: Perceived social support as a potential protective factor. BMJ Open, 7(9). 10.1136/bmjopen-2016-013228. e013228-e013228. PMC5588961 28864684

[R14] Cooper LA, Marsteller JA, Carson KA, Dietz KB, Boonyasai RT, Alvarez C, (2020). The RICH LIFE Project: A cluster randomized pragmatic trial comparing the effectiveness of health system only vs. health system Plus a collaborative/stepped care intervention to reduce hypertension disparities. American Heart Journal, 226, 94–113. 10.1016/j.ahj.2020.05.001 32526534 PMC7481013

[R15] Cornwell EY, & Waite LJ (2012). Social network resources and management of hypertension. Journal of Health and Social Behavior, 53(2), 215–231. 10.1177/0022146512446832 22660826 PMC3727627

[R16] Dube SR, Williamson DF, Thompson T, Felitti VJ, & Anda RF (2004). Assessing the reliability of retrospective reports of adverse childhood experiences among adult HMO members attending a primary care clinic. Child Abuse & Neglect, 28(7), 729–737. 10.1016/j.chiabu.2003.08.009 15261468

[R17] Dvir Y, Ford JD, Hill M, & Frazier JA (2014). Childhood maltreatment, emotional dysregulation, and psychiatric comorbidities. Harvard Review of Psychiatry, 22(3), 149. 24704784 10.1097/HRP.0000000000000014 PMC4091823

[R18] Ekers D, Webster L, Van Straten A, Cuijpers P, Richards D, & Gilbody S. (2014). Behavioural activation for depression; an update of meta-analysis of effectiveness and sub group analysis. PLoS One, 9(6), Article e100100. 10.1371/journal.pone.0100100 PMC4061095 24936656

[R19] Evans SE, Steel AL, & DiLillo D. (2013). Child maltreatment severity and adult trauma symptoms: Does perceived social support play a buffering role? Child Abuse & Neglect, 37(11), 934–943. 10.1016/j.chiabu.2013.03.005 23623620 PMC3758446

[R20] F4analyse. n.d https://www.audiotranskription.de/english/f4-analyse.

[R21] Felitti VJ, Anda RF, Nordenberg D, Williamson DF, Spitz AM, Edwards V, … Marks JS (2019). Relationship of childhood abuse and household dysfunction to many of the leading causes of death in adults: The adverse childhood experiences (ACE) study. American Journal of Preventive Medicine, 56(6), 774–786. 10.1016/J.AMEPRE.2019.04.001 31104722

[R22] Finkelhor D, Shattuck A, Turner H, & Hamby S. (2015). A revised inventory of adverse childhood experiences. Child Abuse & Neglect, 48, 13–21. 26259971 10.1016/j.chiabu.2015.07.011

[R23] Fryar CD, Ostchega Y, Hales CM, Zhang G, & Kruszon-Moran D. (2017), 289. Hypertension prevalence and control among adults: United States, 2015–2016 (pp. 1–8). NCHS data brief. Retrieved from http://www.ncbi.nlm.nih.gov/pubmed/29155682. 29155682

[R24] Godoy LC, Frankfurter C, Cooper M, Lay C, Maunder R, & Farkouh ME (2021). Association of adverse childhood experiences with cardiovascular disease later in life: A review. JAMA cardiology, 6(2), 228–235. 33263716 10.1001/jamacardio.2020.6050

[R25] Hager AD, & Runtz MG (2012). Physical and psychological maltreatment in childhood and later health problems in women: An exploratory investigation of the roles of perceived stress and coping strategies. Child Abuse & Neglect, 36(5), 393–403. 10.1016/J.CHIABU.2012.02.002 22609072

[R26] Health Resources and Services Administration. (2019). Health center program. Available at: https://bphc.hrsa.gov/sites/default/files/bphc/about/healthcenterfactsheet.pdf. (Accessed 22 January 2020).

[R27] Hibbard JH, Greene J, & Overton V. (2013). Patients with lower activation associated with higher costs; delivery systems should know their patients’ ‘scores’. Health Affairs, 32(2), 216–222. 10.1377/hlthaff.2012.1064 ([doi]). 23381513

[R28] Hill TD, Kaplan LM, French MT, & Johnson RJ (2010). Victimization in early life and mental health in adulthood. Journal of Health and Social Behavior, 51(1), 48–63. 10.1177/0022146509361194 20420294

[R29] Hobfoll SE, Johnson RJ, Ennis N, & Jackson AP (2003). Resource loss, resource gain, and emotional outcomes among inner city women. Journal of Personality and Social Psychology, 84(3), 632–643. 10.1037/0022-3514.84.3.632 12635922

[R30] Hooper LM, Tomek S, Roter D, Carson KA, Mugoya G, & Cooper LA (2016). Depression, patient characteristics, and attachment style: correlates and mediators of medication treatment adherence in a racially diverse primary care sample. Primary Health Care Research & Development, 17(2), 184–197. 26810770 10.1017/S1463423615000365

[R31] Irish L, Kobayashi I, & Delahanty DL (2010). Long-term physical health consequences of childhood sexual abuse: A meta-analytic review. Journal of Pediatric Psychology, 35(5), 450–461. 20022919 10.1093/jpepsy/jsp118 PMC2910944

[R32] Jones TM, Nurius P, Song C, & Fleming CM (2018). Modeling life course pathways from adverse childhood experiences to adult mental health. Child Abuse & Neglect, 80, 32–40. 10.1016/J.CHIABU.2018.03.005 29567455 PMC5953821

[R33] Lazarus RS (1993). From psychological stress to the emotions: A history of changing outlooks. Annual Review of Psychology, 44(1), 1–22. 10.1146/annurev.ps.44.020193.000245 8434890

[R34] Lazarus RS, & Folkman S. (1987). Transactional theory and research on emotions and coping. European Journal of Personality, 1(3), 141–169.

[R35] Lewis LM, Askie P, Randleman S, & Shelton-Dunston B. (2010). Medication adherence beliefs of community-dwelling hypertensive African Americans. Journal of Cardiovascular Nursing, 25(3), 199–206. 20386242 10.1097/JCN.0b013e3181c7ccde

[R36] Logan-Greene P, Green S, Nurius PS, & Longhi D. (2014). Distinct contributions of adverse childhood experiences and resilience resources: A cohort analysis of adult physical and mental health. Social Work in Health Care, 53(8), 776–797. 10.1080/00981389.2014.944251 25255340 PMC4273909

[R37] Lopez MA, & Basco MA (2015). Effectiveness of cognitive behavioral therapy in public mental health: Comparison to treatment as usual for treatment-resistant depression. Administration and Policy in Mental Health and Mental Health Services Research, 42(1), 87–98. 10.1007/s10488-014-0546-4 24692026 PMC4183730

[R38] Mc Elroy S, & Hevey D. (2014). Relationship between adverse early experiences, stressors, psychosocial resources and wellbeing. Child Abuse & Neglect, 38(1), 65–75. 24011494 10.1016/j.chiabu.2013.07.017

[R39] Meng L, Chen D, Yang Y, Zheng Y, & Hui R. (2012). Depression increases the risk of hypertension incidence: A meta-analysis of prospective cohort studies. Journal of Hypertension, 30(5), 842–851. 22343537 10.1097/HJH.0b013e32835080b7

[R40] Merrick MT, Ford DC, Ports KA, Guinn AS, Chen J, Klevens J, … Mercy JA (2019). Vital signs:estimated proportion of adult health problems attributable to adverse childhood experiences and implications for prevention — 25 states, 2015–2017. MMWR. Morbidity & Mortality Weekly Report, 68(44), 999–1005. 10.15585/mmwr.mm6844e1 31697656 PMC6837472

[R41] Mersky JP, Janczewski CE, & Topitzes J. (2017). Rethinking the measurement of adversity. Child Maltreatment, 22(1), 58–68. 10.1177/1077559516679513 27920222

[R42] Miles MB, & Huberman AM (1984). Qualitative data analysis: A sourcebook of new methods. In Qualitative data analysis: A sourcebook of new methods (pp. 312–313). Sage publications.

[R43] Morse JM (2015). Critical analysis of strategies for determining rigor in qualitative inquiry. Qualitative Health Research, 25(9), 1212–1222. 26184336 10.1177/1049732315588501

[R44] Mosley-Johnson E, Garacci E, Wagner N, Mendez C, Williams JS, & Egede LE (2019). Assessing the relationship between adverse childhood experiences and life satisfaction, psychological well-being, and social well-being: United States longitudinal cohort 1995–2014. Quality of Life Research, 28(4), 907–914. 10.1007/s11136-018-2054-6 30467779 PMC6459973

[R45] Neergaard MA, Olesen F, Andersen RS, & Sondergaard J. (2009). Qualitative description–the poor cousin of health research? BMC Medical Research Methodology, 9(1), 52. 19607668 10.1186/1471-2288-9-52 PMC2717117

[R46] Nurius PS, Logan-Greene P, & Green S. (2012). Adverse childhood experiences (ACE) within a social disadvantage framework: Distinguishing unique, cumulative, and moderated contributions to adult mental health. Journal of Prevention & Intervention in the Community, 40(4), 278–290. 10.1080/10852352.2012.707443 22970781 PMC3445037

[R47] Nurius PS, Uehara E, & Zatzick DF (2013). Intersection of stress, social disadvantage, and life course processes: Reframing trauma and mental health. American Journal of Psychiatric Rehabilitation, 16(2), 91–114. 25729337 10.1080/15487768.2013.789688 PMC4343539

[R48] Olsen MH, Angell SY, Asma S, Boutouyrie P, Burger D, Chirinos JA, … Wang JG (2016). A call to action and a lifecourse strategy to address the global burden of raised blood pressure on current and future generations: The lancet commission on hypertension. The Lancet, 388(10060), 2665–2712. 10.1016/S0140-6736(16)31134-5 27671667

[R49] Pearlin LI, Menaghan EG, Lieberman MA, & Mullan JT (1981). The stress process. Journal of Health and Social Behavior, 337–356. 7320473

[R50] Peters RM, & Templin TN (2010). Theory of planned behavior, self-care motivation, and blood pressure self-care. Research and Theory for Nursing Practice, 24(3), 172. 20949834 10.1891/1541-6577.24.3.172 PMC3728772

[R51] Petruccelli K, Davis J, & Berman T. (2019). Adverse childhood experiences and associated health outcomes: A systematic review and meta-analysis. Child Abuse & Neglect, 97. 10.1016/J.CHIABU.2019.104127, 104127–104127. 31454589

[R52] Rohner RP, Khaleque A, & Cournoyer DE (2005). Parental acceptance-rejection: Theory, methods, cross-cultural evidence, and implications. Ethos, 33(3), 299–334. 10.1525/eth.2005.33.3.299

[R53] Ross N, Gilbert R, Torres S, Dugas K, Jefferies P, McDonald S, … Ungar M. (2020). Adverse childhood experiences: Assessing the impact on physical and psychosocial health in adulthood and the mitigating role of resilience. Child Abuse & Neglect, 103, Article 104440. 10.1016/j.chiabu.2020.104440 32135375

[R54] Rubio-Guerra AF, Rodriguez-Lopez L, Vargas-Ayala G, Huerta-Ramirez S, Serna DC, & Lozano-Nuevo JJ (2013). Depression increases the risk for uncontrolled hypertension. Experimental and Clinical Cardiology, 18(1), 10. 24294029 PMC3716493

[R55] Rueda B, & Pérez-García AM (2006). A prospective study of the effects of psychological resources and depression in essential hypertension. Journal of Health Psychology, 11(1), 129–140. 10.1177/1359105306058868 16314386

[R56] Sandelowski M. (2000). Whatever happened to qualitative description? Research in Nursing & Health, 23(4), 334–340. 10940958 10.1002/1098-240x(200008)23:4<334::aid-nur9>3.0.co;2-g

[R57] Saunders B, Sim J, Kingstone T, Baker S, Waterfield J, Bartlam B, Burroughs H, & Jinks C. (2018). Saturation in qualitative research: Exploring its conceptualization and operationalization. Quality and Quantity, 52(4), 1893–1907. 10.1007/s11135-017-0574-8 29937585 PMC5993836

[R58] Scribner RA, Simonsen NR, & Leonardi C. (2017). The social determinants of health core: Taking a place-based approach. American Journal of Preventive Medicine, 52(1), S13–S19. 27989288 10.1016/j.amepre.2016.09.025 PMC6540790

[R59] Spaccarelli S. (1994). Stress, appraisal, and coping in child sexual abuse: A theoretical and empirical review. Psychological Bulletin, 116(2), 340. 10.1037/0033-2909.116.2.340 7972595

[R60] Su S, Jimenez MP, Roberts CTF, & Loucks EB (2015). The role of adverse childhood experiences in cardiovascular disease risk: A review with emphasis on plausible mechanisms. Current Cardiology Reports, 17(10). 10.1007/s11886-015-0645-1, 88–88. PMC4941633 26289252

[R61] Warren-Findlow J, Seymour RB, & Huber LRB (2012). The association between self-efficacy and hypertension self-care activities among African American adults. Journal of Community Health, 37(1), 15–24. 10.1007/s10900-011-9410-6 21547409 PMC3179559

[R62] Wilson DR (2010). Health consequences of childhood sexual abuse. Perspectives in Psychiatric Care, 46(1), 56–64. 20051079 10.1111/j.1744-6163.2009.00238.x

[R63] Wu Y, Levis B, Riehm KE, Saadat N, Levis AW, Azar M, … Ioannidis JP (2019). Equivalency of the diagnostic accuracy of the PHQ-8 and PHQ-9: A systematic review and individual participant data meta-analysis. Psychological Medicine, 1–13.

